# Fatigue, fear of being mobilized and residual limb pain limit independent basic mobility and physiotherapy for patients early after major dysvascular lower extremity amputation: A prospective cohort study

**DOI:** 10.1111/ggi.14874

**Published:** 2024-04-10

**Authors:** Anja Løve Berger, Annie Østergaard Nielsen, Sanne Busk Stie, Morten Tange Kristensen

**Affiliations:** ^1^ Department of Physical and Occupational Therapy Copenhagen University Hospital, Bispebjerg and Frederiksberg Copenhagen Denmark; ^2^ Physical Medicine and Rehabilitation Research‐Copenhagen (PMR‐C), Department of Physical and Occupational Therapy Copenhagen University Hospital, Amager and Hvidovre Hvidovre Denmark; ^3^ Department of Clinical Medicine University of Copenhagen Copenhagen Denmark

**Keywords:** basic mobility, early mobilization, major lower extremity amputation, mobility limitation, physiotherapy

## Abstract

**Aim:**

Early mobilization of patients with a major lower extremity amputation (LEA) is often a challenge because of lack of compliance. Therefore, we investigated factors limiting independent mobility and physiotherapy on the first day with physiotherapy (PTDay1) and the following 2 days after LEA.

**Methods:**

A total of 60 consecutive patients, mean age 73.7 years (SD 12.1 years), undergoing LEA were included over a period of 7 months. The Basic Amputee Mobility Score was used to assess basic mobility. Predefined limitations for not achieving independent mobility or not completing physiotherapy were residual limb pain, pain elsewhere, fear of being mobilized, fatigue, nausea/vomiting, acute cognitive dysfunction or “other” factors reported on PTDay1 and the following 2 days after LEA.

**Results:**

Fatigue and fear of being mobilized were the most frequent limitations for not achieving independent mobility on PTDay1 and the following 2 days after LEA. Patients (*n* = 55) who were not independent in the Basic Amputee Mobility Score activity transferring from bed to chair on PTDay1 were limited by fatigue (44%) and fear of being mobilized (33%). A total of 21 patients did not complete planned physiotherapy on PTDay1, and were limited by fatigue (38%), residual limb pain (24%) and “other” factors (24%).

**Conclusion:**

Fatigue and fear of being mobilized were the most frequent factors that limited independent mobility early after LEA. Fatigue, residual limb pain and “other” factors limited completion of physiotherapy. **Geriatr Gerontol Int 2024; 24: 470–476**.

## Graphical abstract

Fatigue and fear of being mobilized are the most frequent limitations for not achieving independent mobility early after dysvascular lower extremity amputation, whereas fatigue and residual limb pain limit planned physiotherapy.

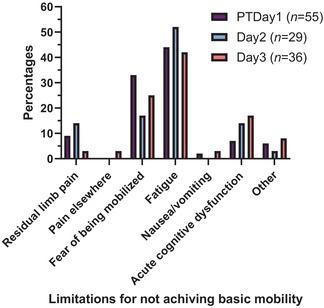

## Introduction

A major dysvascular lower extremity amputation (LEA) is associated with a high risk of mortality,^1^ multimorbidity^2^, ^3^ and loss of physical function.^4^, ^5^ Few LEA patients achieve independent mobility within the first post‐amputation days, and this increases to approximately 50% at discharge from the acute hospital.^6^ Studies have shown that early mobilization out of bed starting as early as on the first postoperative day for patients with LEA seems to increase physical function,^8^, ^9^, ^10^, ^11^ and reduce mortality and complications.^6^, ^7^ Yet, early mobilization is complex and not achievable for all LEAs.^6^ Thus, identifying factors that limit early mobilization and the ability to achieve independent mobility after LEA is essential for planning effective strategies to enhance recovery. In comparison, factors that limit early mobilization and participation in physiotherapy have been established for patients with hip fracture^12^ and after acute high‐risk abdominal surgery,^13^, ^14^ but to our knowledge, not after LEA.

The primary aim was to examine the most frequent factors limiting independent mobility during the first postoperative days after a major dysvascular LEA. Second, to examine factors that limit completion of planned physiotherapy on the first day with physiotherapy (PTDay1) and the following 2 days (Day2‐3), in addition to exploring the association between the most frequent limiting factors and patient characteristics.

## Methods

### Study design and patients

A prospective observational cohort study of patients who consecutively underwent a major dysvascular LEA at the Copenhagen University Hospital Hvidovre, Denmark, was carried out between mid‐November 2018 and June 2019. Inclusion criteria were objectively assessed with the Basic Amputee Mobility Score (BAMS)^6^ on PTDay1. Patients provided informed consent before participation. Exclusion criteria were residual limb revision, and when a patient had not received physiotherapy during admittance.

The study was approved by the local ethics committee (wz19001024‐2019‐14). Data collection was registered with the regional data protection agency (J.no.01 HVH‐2012‐053). The study adheres to the STROBE checklist.^15^

### Treatment program

All patients followed an enhanced multimodal perioperative program adapted to patients with a major LEA, standardized for elements, such as rehabilitation, blood transfusion and pain management.^7^

The program encompasses the initiation of physiotherapy on PTDay1 regardless of what day of the week. Thereafter, physiotherapy was only offered on weekdays. Thus, in the present study, data for Day2‐3 only includes those patients offered physiotherapy on three consecutive days. The physiotherapy sessions are focused on patients achieving independent basic mobility, increasing balance/muscle strength and possibly preparing the patient for a prosthesis. This includes training in getting from a supine position to sitting, transfer, use of assistive devices and wheelchair maneuvering, and progressed on an individual level.

### The Basic Amputee Mobility Score

The BAMS was used for the daily assessment of basic mobility,^6^ and is defined by four activities: (1) from supine lying in bed to sitting on the edge of the bed and back, (2) from sitting on the edge of the bed to a chair/wheelchair and back, (3) indoor wheelchair mobility, and (4) from a chair/wheelchair to standing and back. Each of the four activities was scored from 0 to 2 by one of two experienced physiotherapists, where “0 = not able to despite extensive help from one or more persons, 1 = able to with verbal instruction to extensive help from one or more persons, and 2 = able to safely, without verbal instruction or support from a person, even for safety reasons”. It provides a daily cumulated BAMS score of 0–8, with 8 points being equivalent to the patient having independent basic mobility.

BAMS is utilized in several Danish and international hospitals. It has shown excellent inter‐rater reliability (Kappa 0.98), high responsiveness, validity for known age groups and proven as a strong predictor of 30‐day mortality after LEA.^6^

### Outcomes and assessments

The following patient demographics were recorded: sex, age, the American Society of Anesthesiologists grade (1–4) of physical status,^16^ amputation level, the final amputation status (transtibial‐, transfemoral‐ or bilateral), primary diagnosis, primary reason for amputation, number of days since amputation until PTDay1 and weekday of surgery. On discharge, the length of stay and discharge destination (another ward, own residence/nursing home or temporary 24‐h setting in municipality) were recorded. Mortality during admittance, after 30 days, and 6 months were verified by national register data.

### Factors limiting basic mobility and physiotherapy

Patients were BAMS scored by the physiotherapist, and those unable to carry out one or more of the four BAMS activities independently, equal to a score of 0 or 1, were considered limited in their basic mobility. For activities with a BAMS <2 points, the primary and possibly a secondary factor reported by the patient was recorded from a set of predefined limitations (Table S1). Likewise, a primary and possibly a secondary factor limiting physiotherapy was reported by the patient if unable to complete the planned physiotherapy. In rare cases, patients were unable to report a limitation, in which case the physiotherapist did so to the best of their ability.

The factors limiting independent basic mobility and physiotherapy were predefined by experienced amputee physiotherapists at the hospital, and were based on consensus discussions and inspiration from a similar study.^12^ The predefined limiting factors were: (1) residual limb pain, (2) pain elsewhere, (3) fear of being mobilized, (4) fatigue, (5) nausea/vomiting, (6) acute cognitive dysfunction (delirium), or (7) other limitations. In the cases where the limitation was “other,” the patient individually defined the limitation, and it was recorded on the scoring sheet (Table S1).

### Physical therapy

After each physiotherapy session, the physiotherapist assessed whether the patient had fully completed, partially completed or not completed the planned physiotherapy using a method from a similar study.^12^ One of the three options was noted on the scoring sheet with Y/P/N, where Y = Yes when the patient had completed the session, P = Partially completed when the patient had only been able to participate partially or had to give up exercises and N = Not completed. The same predefined factors limiting mobility were used for patients not able to fully complete the planned physiotherapy.

### Statistical analysis

Data were examined for normality of distribution using the Kolmogorov–Smirnoff test. The study population was described by the mean and standard deviation when normally distributed, and otherwise by median and 25%–75% quartiles, or as numbers with percentages, as appropriate. The BAMS score, completion of physiotherapy on PTDay1 and Day2‐3, and the most frequent primary limitations on PTDay1 and Day2‐3 for not achieving a BAMS score of 2 per activity and not completing the planned physiotherapy, were described with numbers (*n*) and percentages. Very few secondary limitations (Table S2) were reported by patients for both assessments, and therefore not included in results and analysis. Analyses for factors limiting independent basic mobility were a priori focused on BAMS activity 2, transferring from bed to chair/wheelchair and back. This activity is particularly challenging for patients with LEA, and provides knowledge of whether the patient has been mobilized out of bed or not.

The χ^2^‐test was used to evaluate the association between the most frequent limitations for BAMS activity 2 on PTDay1 and for the following subgroups: sex (male *vs* female), age (≤74 years *vs* >74 years), American Society of Anesthesiologists grade score (2–3 *vs* 4), the final amputation status (transtibial *vs* transfemoral/bilateral) and the primary diagnosis (diabetes mellitus *vs* atherosclerosis/other).

Statistical analyses were carried out using SPSS version 25 (IBM Corporation, Armonk, NY, USA), and the level of statistical significance was set at *P* < 0.05. Figures were developed using GraphPad Prism 10.1 (San Diego, CA, USA).

## Results

During the 7‐month recruitment period, a total of 70 patients underwent LEA at the hospital, of whom four were excluded due to stump revision surgery, four died before PTDay1, one had missing data and one had not received physiotherapy during admittance leaving 60 included patients.

The characteristics of the study population are presented in Table 1. PTDay1 occurred on the first postoperative day for 55 (91.7%) patients, and on day 2 for three patients (5%); one due to late return to the ward after surgery, and two for unknown reasons. For two patients (3.3%), PTDay1 was on postoperative day 4 due to an intensive care unit stay for 3 days after amputation.

**Table 1:** Characteristics of the study population with a major dysvascular lower extremity amputation

Variables	*n* (%)
Mean age, years (SD)	73.7 (12.1)
Women	25 (41.7)
Men	35 (58.3)
ASA grade, 1–4 points	
Grade 2	5 (8.3)
Grade 3	42 (70)
Grade 4	13 (21.7)
Present amputation level	
Transfemoral	40 (66.7)
Transtibial	19 (31.7)
Bilateral	1 (1.7)
Final amputation status	
Transfemoral	36 (60)
Transtibial	17 (28.3)
Bilateral	7 (11.7)
Primary diagnosis	
Diabetes mellitus	29 (48.3)
Arteriosclerosis	26 (43.3)
Other^†^	5 (8.3)
Primary cause of amputation	
Infection/wound	30 (50)
Gangrene	22 (36.7)
Complications to surgery	5 (8.3)
Thrombosis/embolism	2 (3.3)
Other^‡^	1 (1.7)
Day of amputation	
Monday	5 (8.3)
Tuesday	17 (28.3)
Wednesday	6 (10)
Thursday	3 (5)
Friday	21 (35)
Saturday	4 (6.7)
Sunday	4 (6.7)
No. days from amputation to PTDay1, median (25%–75% quartiles)	1 (1–1)
On the day of amputation	1 (1.7)
Day 1 after amputation	54 (90)
Day 2 after amputation	3 (5)
Day 4 after amputation	2 (3.3)
Discharge destination	
Other wards	4 (7.4)
Own home/nursing home	24 (44.4)
Temporary 24‐h setting in municipality	26 (48.1)
Mean length of stay in, days (SD)	12.74 (5.3)
Mortality within admittance	5 (8.3)
Mortality, 30 days post‐amputation	12 (20)
Mortality, 6 months post‐amputation	16 (27.6)

Total *n* = 60. Variables are presented as number of patients (percentages) or as indicated.

^†^ Embolism, paraplegia, cancer, hypertension.

^‡^ Ischemic pain without ulceration.

ASA: American Society of Anesthesiologists grade. PTDay1: first post‐amputation day with physiotherapy.

### Basic mobility and its limitations

The frequency of BAMS scores for the four activities separately on PTDay1 and Day2‐3 are presented in Table 2. Only three patients (5%) had an independent basic mobility score of 2 on all four BAMS activities on PTDay1. This was unchanged on day 2, whereas it increased to six patients (10%) on day 3.

**Table 2:** Frequency of the Basic Amputee Mobility Score (BAMS, 0–8 points) on three consecutive days after a major lower extremity amputation

Day	BAMS activity 1			BAMS activity 2			BAMS activity 3			BAMS activity 4^†^		
	From supine lying in bed to sitting on the edge of the bed and back			From sitting on the edge of the bed to a chair/wheelchair and back			Indoor wheelchair mobility			From chair/wheelchair to standing on the non‐amputated leg and back		
	BAMS 0–2 points			BAMS 0–2 points			BAMS 0–2 points			BAMS 0–2 points		
	0	1	2	0	1	2	0	1	2	0	1	2
PTDay1 (*N* = 60)	21 (35)	24 (40)	15 (25)	13 (21.7)	42 (70)	5 (8.3)	14 (23.3)	31 (51.7)	15 (25)	46 (78)	9 (15.3)	4 (6.8)
Day2 (*n* = 34)^‡^	9 (26.5)	18 (52.9)	7 (20.6)	4 (11.8)	25 (73.5)	5 (14.7)	5 (14.7)	20 (58.8)	9 (26.5)	24 (70.6)	7 (20.6)	3 (8.8)
Day3 (*n* = 44)^‡^	14 (31.8)	17 (38.6)	13 (29.5)	9 (20.5)	27 (61.4)	8 (18.2)	9 (20.5)	17 (38.6)	18 (40.9)	27 (62.8)	9 (20.9)	7 (16.3)

Total *n* = 60. Data are shown as the number (%). PTDay1 and Day2‐3: the first day with physiotherapy and the following 2 days. BAMS score: 0 = not possible, 1 = possible with assistance, 2 = independent.

^†^ One patient had missing data on activity 4 on all 3 days.

^‡^ Missing data due to patients not receiving any physiotherapy on weekends and public holidays after postoperative day 1, and due to staff logistics.

The number of patients evaluated with the BAMS on Day2‐3 was lower than on PTDay1, mainly because many patients underwent LEA on a Friday (35%; Table 1), and as physiotherapy is only offered during weekends on postoperative day 1.

**Figure 1:**
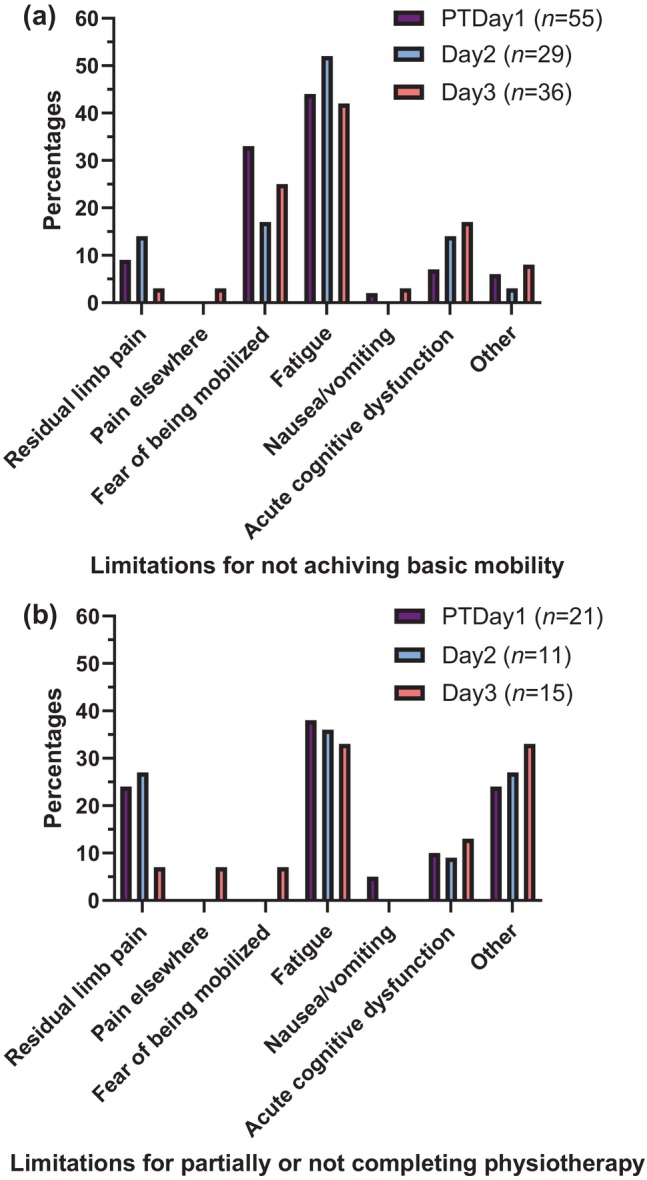
(a) Limitations for not achieving an independent Basic Amputee Mobility Score (BAMS) of 2 points for activity 2: getting from bed to wheelchair and back, on the first day with physiotherapy (PTDay1) and the two following days (Day2‐3), and (b) limitations for partially or not completing planned physiotherapy on the corresponding days.

Fatigue and fear of being mobilized were the most frequent factors limiting all four BAMS activities on all 3 days, and followed by residual limb pain and acute cognitive dysfunction (Table 3). The results for BAMS activity 2 (transfer from bed to chair and back) are shown in Figure 1a. The factor, “other limitations,” for non‐independency in activity 2 was reported as problematic blood pressure/blood sugar, unconsciousness or not motivated. Overall, the patients primarily utilized the predefined factors limiting them, and rarely the individually defined “other” option. Likewise, only in very rare cases did the physiotherapist have to decide the factor limiting patients.

**Table 3:** Limitations for not achieving an independent Basic Amputee Mobility Score of 2 points for each of the four Basic Amputee Mobility Score activities on three consecutive days after major lower extremity amputation

	PTDay1, *N* = 60				Day2, *n* = 34^†^				Day3, *n* = 44^†^			
Limiting factors	A1	A2	A3	A4	A1	A2	A3	A4	A1	A2	A3	A4
	(*n* = 45)	(*n* = 55)	(*n* = 44)	(*n* = 56)	(*n* = 27)	(*n* = 29)	(*n* = 25)	(*n* = 31)	(*n* = 31)	(*n* = 36)	(*n* = 26)	(*n* = 37)
Residual limb pain	4 (8.9)	5 (9.1)	2 (4.5)	4 (7.1)	3 (11.1)	4 (13.8)	1 (4)	3 (9.7)	2 (6.5)	1 (2.8)	1 (3.8)	‐
Pain elsewhere	‐	‐	‐	‐	‐	‐	‐	‐	‐	1 (2.8)	1 (3.8)	1 (13.5)
Fear of being mobilized	9 (20)	18 (32.7)	1 (2.3)	7 (12.5)	5 (18.5)	5 (17.2)	1 (4)	3 (9.7)	7 (22.6)	9 (25)	1 (3.8)	5 (13.5)
Fatigue	24 (53.3)	24 (43.6)	32 (72.7)	28 (50)	14 (51.9)	15 (51.7)	17 (68)	17 (54.8)	13 (41.9)	15 (41.7)	13 (50)	19 (51.4)
Nausea/vomiting	1 (2.2)	1 (1.8)	1 (2.3)	1 (1.8)	‐	‐	‐	‐	‐	1 (2.8)	‐	‐
Acute cognitive dysfunction	4 (8.9)	4 (7.3)	4 (9.1)	4 (7.1)	4 (14.8)	4 (13.8)	4 (16)	4 (12.9)	6 (19.4)	6 (16.7)	6 (23.1)	6 (16.2)
Other	3 (6.7)	3 (5.5)	4 (9.1)	12 (21.4)	1 (3.7)	1 (3.4)	2 (8)	4 (12.9)	3 (9.7)	3 (8.3)	4 (15.4)	6 (16.2)

Total *n* = 60. Data are shown as numbers (%). PTDay1 and Day2‐3: the first day with physiotherapy and the following 2 days. A1–A4: Basic Amputee Mobility Score Activity 1–4. A1. from supine lying in bed to sitting on the edge of the bed and back, A2. from sitting on the edge of the bed to a chair/wheelchair and back, A3. indoor wheelchair mobility and A4. from a chair/wheelchair to standing and back.

^†^ Missing data due to patients not receiving any physiotherapy on weekends and public holidays after postoperative day one, and due to staff logistics.

There was no significant association between fatigue or fear of being mobilized as a limitation for BAMS activity 2 on PTDay1 and the previously described subgroups for sex, age, American Society of Anesthesiologists grade, the final amputation status, and the primary diagnosis.

### Planned physiotherapy and its limitations

A total of 36, 22 and 29 patients completed the planned physiotherapy on PTDay1, day 2 and day 3, respectively (Table 4). Fatigue, residual limb pain and “other” were the factors that most often limited patients when only partially completing or not completing planned physiotherapy on PTDay1 and Day2‐3 (Table 4, Figure 1B). The factor, “other limitations,” was problematic blood pressure/blood sugar, unconsciousness, patient not available or not motivated.

**Table 4:** Planned physiotherapy, completed, partially completed, or not completed, and limitations for those not completed on three consecutive days after major lower extremity amputation

	PTDay1 (*N* = 60)	Day2 (*n* = 57)^†^	Day3 (*n* = 58)^†^
Completion of planned physiotherapy			
Yes	36 (63)	22 (67)	29 (66)
Partially	5 (9)	5 (15)	1 (2)
No	16 (28)	6 (18)	14 (32)
Physiotherapy not offered^‡^	3	24	14

	PTDay1 (*n* = 21)	Day2 (*n* = 11)	Day3 (*n* = 15)
Limitations for partially or not completed planned physiotherapy			
Residual limb pain	5 (23.8)	3 (27.3)	1 (6.7)
Pain elsewhere	‐	‐	1 (6.7)
Fear of being mobilized	‐	‐	1 (6.7)
Fatigue	8 (38.1)	4 (36.4)	5 (33.3)
Nausea/vomiting	1 (4.8)	‐	‐
Acute cognitive dysfunction	2 (9.5)	1 (9.1)	2 (13.3)
Other	5 (23.8)	3 (27.3)	5 (33.3)

Total *n* = 60. Data are shown as numbers (%). PTDay1 and Day2‐3: the first day with physiotherapy and the following 2 days. Other: Not able to collaborate, not motivated, low blood pressure, low blood sugar, and unconsciousness.

^†^ Missing data for unknown reasons.

^‡^ Physiotherapy not offered due to weekends, public holidays, dementia, discharge, transfer to another ward and poor condition.

## Discussion

The present study shows that fatigue and fear of being mobilized are the most frequent reasons for patients not achieving independent basic mobility early after a major dysvascular LEA. Additionally, we found that fatigue, residual limb pain and “other” limitations are the most frequent reasons for not completing planned physiotherapy. By gaining this knowledge of what patients perceived as limiting their basic activities, clinicians have a better understanding of the limited resources and challenges that these frail patients are facing.

To our knowledge, this is the first study to describe patient‐reported factors limiting independent mobility and participation in physiotherapy in the important first days after a major dysvascular LEA. It thereby adds valuable knowledge to further enhance the early rehabilitation and potentially improve outcomes of this frail patient group. The current findings are consistent with studies of patients after acute abdominal surgery^14^ and patients with hip fracture,^12^, ^17^ which to some extent can be considered a patient group with similar frailty.

We cannot conclude on the cause of fatigue in the present study. However, it is noteworthy that >90% of the patients had a primary diagnosis of either diabetes or atherosclerosis, which are conditions associated with physical and mental fatigue,^18^ and peripheral fatigue.^19^ Additionally, postoperative fatigue, a well‐known condition after surgery,^20^ is a plausible explanation for fatigue being a major limitation in the present study. Furthermore, anemia is a common condition among patients with a major LEA,^21^ and is likewise associated with fatigue in older and weakened populations,^22^, ^23^ that might limit the ability to recover from surgery and participate in rehabilitation.^24^, ^25^ In patients with hip fracture,^26^ anemia has been associated with a low ambulatory level in the first three postoperative days, and the same is plausible for patients in the present study.

The second most frequent factor limiting independent basic mobility was fear of being mobilized, reported by 33% of patients who were not able to independently transfer from bed to wheelchair on PTDay1. The present findings align with previous research, indicating that patients with LEA commonly experience fear of falling not only within the first year, but also several years after LEA,^27^ and fear of falling can be a barrier to physical activity.^28^ Extremely poor one‐leg balance performance has also been reported early after dysvascular LEA,^6^, ^29^ and according to Miller *et al*., patients with LEA often have a low balance confidence immediately after LEA, as well as several years thereafter.^30^, ^31^, ^32^ Among patients who underwent LEA several years earlier, low balance confidence is associated with reduced mobility^33^, ^34^ and a lower degree of daily physical activity.^35^ A better understanding of fear avoidance and how it influences early mobilization after LEA is needed, and the findings of this study can serve as a backdrop for future strategies to address this issue.

Residual limb pain, which was the second most frequent factor limiting the completion of planned physiotherapy, corresponds well with findings in other patient groups that report pain as a frequent limitation for early mobilization and exercise during hospitalization.^12^, ^36^, ^37^, ^38^ Likewise, the prevalence of residual limb pain is high shortly after and at long‐term follow up in LEAs.^39^, ^40^ Together, this highlights the importance of giving high priority to effective pain management in acute care settings after LEA. The variety of “other” limitations highlights the complexity of LEAs and the many factors that must be considered to achieve successful early mobilization.

Contrary to findings of previous studies, we did not find a significant association between the most frequent limitations and patient characteristics on the activity transfer from bed to wheelchair. Thus, a positive association has been reported between postoperative fatigue and age in patients after elective abdominal surgery^41^ and gastrointestinal tumor surgery.^42^ Also, the percentage of older people who experience fatigue in daily activities seems to increase significantly between 70 and 85 years‐of‐age, and can be related to loss of functional ability.^43^ This was not the case in the present study. Fear of falling is also associated with older age among older adults,^44^, ^45^ but was also not the case in this study.

We also expected more transfemoral/bilateral amputees than transtibial amputees to be limited by fear of being mobilized. This assumption stemmed from the greater challenge individuals with a transfemoral LEA might face in maintaining balance due to the absence of both the ankle and knee joint, in contrast to those with a transtibial amputation.^31^, ^46^ However, contrary to our expectations, this was not the case. Particularly regarding bilateral amputees, this observation might be linked to many of them already having experience in functioning with one leg before their current amputation.

Strengths of the present study are the use of BAMS as a valid and reliable outcome measure developed and recommended for use in LEAs,^47^ and the representative sample of patients. With an annual incidence of approximately 1800 major LEAs in Denmark, 60 patients from a single hospital for a period of 7 months can be considered representative.^48^ Other strengths are the predefined limiting factors defined by experienced amputee physiotherapists, and that patients were included consecutively, regardless of previous functional and cognitive status, strengthening the results' generalizability. However, a limitation might be that the development of the predefined factors did not directly involve patients with a former LEA. The presence of selection bias cannot be ruled out, as a predefined limitation might inadvertently influence the patients toward choosing from the already listed limitations. However, patients had the opportunity to report the factors limiting their abilities whenever they felt it necessary, although they seldom did. This indicates that the predefined factors might adequately cover the patient‐perceived limitations for mobilization.

Most patients undergoing a major dysvascular LEA were unable to achieve independent basic mobility during the early postoperative period, and were most often restricted by fatigue and fear of being mobilized. Correspondingly, fatigue, residual limb pain and “other” were the most frequent factors that limited completion of planned physiotherapy.

We suggest that efforts to reduce the influence of the identified patient‐reported factors limiting early mobilization and physiotherapy should be considered in acute care settings to potentially increase compliance and ensure better recovery.

## Authors contributions

MTK, AØN and SBS designed the study. ALB carried out the analysis and drafted the work in consultation with MTK. AØN and SBS revised it, and all authors approved the final version.

## Disclosure statement

MTK is the inventor of BAMS, but did not participate in data collection. AØN participated in development of BAMS, but not in analysis. The other authors declare no conflict of interest.

## Supporting information


**Table S1.** Basic Amputee Mobility Score and planned Physiotherapy scoring sheet.

**Table S2.** Secondary limitations for not achieving an independent Basic Amputee Mobility Score (BAMS) of 2 points for each of the 4 BAMS activities and for partially or not completed planned physiotherapy on three consecutive days after major lower extremity amputation, *N* = 60.

## Data Availability

The data that support the findings of this study are available from the corresponding author upon reasonable request.

## References

[1] Kristensen MT , Holm G , Kirketerp‐Møller K *et al*. Very low survival rates after non‐traumatic lower limb amputation in a consecutive series: what to do? Interact Cardiovasc Thorac Surg 2012; 14: 543–547. 22298857 10.1093/icvts/ivr075 PMC3329303

[2] Jensen PS , Petersen J , Kirketerp‐Møller K , Poulsen I , Andersen O . Progression of disease preceding lower extremity amputation in Denmark: a longitudinal registry study of diagnoses, use of medication and healthcare services 14 years prior to amputation. BMJ Open 2017; 7: e016030. 10.1136/bmjopen-2017-016030 PMC5695421 29101132

[3] Sureshkumar A , Payne MW , Viana R , Hunter SW . An eight‐year analysis of participant characteristics at admission to inpatient prosthetic rehabilitation following a lower limb amputation: a Canadian perspective. Disabil Rehabil 2023; 1‐11: 1–11. 10.1080/09638288.2023.2240231 37498002

[4] Madsen UR , Hommel A , Berthelsen CB , Bååth C . Systematic review describing the effect of early mobilisation after dysvascular major lower limb amputations. J Clin Nurs 2017; 26: 3286–3297. 28042882 10.1111/jocn.13716

[5] Frykberg RG , Arora S , Pomposelli FB Jr , LoGerfo F . Functional outcome in the elderly following lower extremity amputation. J Foot Ankle Surg 1998; 37: 181–185 discussion 261, 185. 9638540 10.1016/s1067-2516(98)80107-5

[6] Kristensen MT , Nielsen A , Topp UM *et al*. Development and psychometric properties of the basic amputee mobility score for use in patients with a major lower extremity amputation. Geriatr Gerontol Int 2018; 18: 138–145. 28858422 10.1111/ggi.13156

[7] Kristensen MT , Holm G , Krasheninnikoff M , Jensen PS , Gebuhr P . An enhanced treatment program with markedly reduced mortality after a transtibial or higher non‐traumatic lower extremity amputation. Acta Orthop 2016; 87: 306–311. 27088484 10.3109/17453674.2016.1167524 PMC4900091

[8] O'Banion LA , Dirks R , Farooqui E *et al*. Outcomes of major lower extremity amputations n dysvascular patients: room for improvement. Am J Surg 2020; 220: 1506–1510. 32891397 10.1016/j.amjsurg.2020.08.020

[9] Marzen‐Groller KD , Tremblay SM , Kaszuba J *et al*. Testing the effectiveness of the amputee mobility protocol: a pilot study. J Vasc Nurs 2008; 26: 74–81. 18707996 10.1016/j.jvn.2008.05.001

[10] Schon LC , Short KW , Soupiou O , Noll K , Rheinstein J . Benefits of early prosthetic management of transtibial amputees: a prospective clinical study of a prefabricated prosthesis. Foot Ankle Int 2002; 23: 509–514. 12095119 10.1177/107110070202300607

[11] Ali MM , Loretz L , Shea A *et al*. A contemporary comparative analysis of immediate postoperative prosthesis placement following below‐knee amputation. Ann Vasc Surg 2013; 27: 1146–1153. 23972636 10.1016/j.avsg.2012.10.031

[12] Münter KH , Clemmesen CG , Foss NB , Palm H , Kristensen MT . Fatigue and pain limit independent mobility and physiotherapy after hip fracture surgery. Disabil Rehabil 2018; 40: 1808–1816. 28415885 10.1080/09638288.2017.1314556

[13] Jønsson LR , Foss NB , Orbæk J , Lauritsen ML , Sejrsen HN , Kristensen MT . Early intensive mobilization after acute high‐risk abdominal surgery: a nonrandomized prospective feasibility trial. Can J Surg 2023; 66: E236–E245. 37130709 10.1503/cjs.008722 PMC10158751

[14] Jønsson LR , Ingelsrud LH , Tengberg LT , Bandholm T , Foss NB , Kristensen MT . Physical performance following acute high‐risk abdominal surgery: a prospective cohort study. Can J Surg 2018; 61: 42–49. 29368676 10.1503/cjs.012616 PMC5785288

[15] von Elm E , Altman DG , Egger M , Pocock SJ , Gøtzsche PC , Vandenbroucke JP . The strengthening the reporting of observational studies in epidemiology (STROBE) statement: guidelines for reporting observational studies. J Clin Epidemiol 2008; 61: 344–349. 18313558 10.1016/j.jclinepi.2007.11.008

[16] Mayhew D , Mendonca V , Murthy BVS . A review of ASA physical status—historical perspectives and modern developments. Anaesthesia 2019; 74: 373–379. 30648259 10.1111/anae.14569

[17] Kronborg L , Bandholm T , Palm H , Kehlet H , Kristensen MT . Feasibility of progressive strength training implemented in the acute ward after hip fracture surgery. PloS One 2014; 9: e93332. 24699276 10.1371/journal.pone.0093332 PMC3974729

[18] Kalra S , Sahay R . Diabetes Fatigue Syndrome. Diabetes Ther 2018; 9: 1421–1429. 29869049 10.1007/s13300-018-0453-x PMC6064586

[19] Schainfeld RM . Management of peripheral arterial disease and intermittent claudication. J Am Board Fam Pract 2001; 14: 443–450. 11757887

[20] Zargar‐Shoshtari K , Hill AG . Postoperative fatigue: a review. World J Surg 2009; 33: 738–745. 19189174 10.1007/s00268-008-9906-0

[21] Desormais I , Aboyans V , Bura A *et al*. Anemia, an independent predictive factor for amputation and mortality in patients hospitalized for peripheral artery disease. Eur J Vasc Endovasc Surg 2014; 48: 202–207. 24935912 10.1016/j.ejvs.2014.04.005

[22] Yellen SB , Cella DF , Webster K , Blendowski C , Kaplan E . Measuring fatigue and other anemia‐related symptoms with the functional assessment of cancer therapy (FACT) measurement system. J Pain Symptom Manage 1997; 13: 63–74. 9095563 10.1016/s0885-3924(96)00274-6

[23] Ranucci M , La Rovere MT , Castelvecchio S *et al*. Postoperative anemia and exercise tolerance after cardiac operations in patients without transfusion: what hemoglobin level is acceptable? Ann Thorac Surg 2011; 92: 25–31. 21592458 10.1016/j.athoracsur.2011.02.058

[24] Carson JL , Terrin ML , Jay M . Anemia and postoperative rehabilitation. Can J Anaesth 2003; 50: S60–S64. 14629055

[25] Partridge J , Harari D , Gossage J , Dhesi J . Anaemia in the older surgical patient: a review of prevalence, causes, implications and management. J R Soc Med 2013; 106: 269–277. 23759887 10.1177/0141076813479580 PMC3704065

[26] Foss NB , Kristensen MT , Kehlet H . Anaemia impedes functional mobility after hip fracture surgery. Age Ageing 2008; 37: 173–178. 18349013 10.1093/ageing/afm161

[27] Miller WC , Speechley M , Deathe B . The prevalence and risk factors of falling and fear of falling among lower extremity amputees. Arch Phys Med Rehabil 2001; 82: 1031–1037. 11494181 10.1053/apmr.2001.24295

[28] Littman AJ , Boyko EJ , Thompson ML , Haselkorn JK , Sangeorzan BJ , Arterburn DE . Physical activity barriers and enablers in older veterans with lower‐limb amputation. J Rehabil Res Dev 2014; 51: 895–906. 25356624 10.1682/JRRD.2013.06.0152

[29] Kristensen MT , Nielsen A , Topp UM *et al*. Number of test trials needed for performance stability and interrater reliability of the one leg stand test in patients with a major non‐traumatic lower limb amputation. Gait Posture 2014; 39: 424–429. 24021523 10.1016/j.gaitpost.2013.08.017

[30] Miller WC , Deathe AB . The influence of balance confidence on social activity after discharge from prosthetic rehabilitation for first lower limb amputation. Prosthet Orthot Int 2011; 35: 379–385. 21846808 10.1177/0309364611418874

[31] Miller WC , Speechley M , Deathe AB . Balance confidence among people with lower‐limb amputations. Phys Ther 2002; 82: 856–865. 12201800

[32] Miller W , Deathe A . A prospective study examining balance confidence among individuals with lower limb amputation. Disabil Rehabil 2004; 26: 875–881. 15497916 10.1080/09638280410001708887

[33] Sions JM , Manal TJ , Horne JR , Sarlo FB , Pohlig RT . Balance‐confidence is associated with community participation, perceived physical mobility, and performance‐based function among individuals with a unilateral amputation. Physiother Theory Pract 2020; 36: 607–614. 29952694 10.1080/09593985.2018.1490939 PMC6310658

[34] Miller WC , Deathe AB , Speechley M , Koval J . The influence of falling, fear of falling, and balance confidence on prosthetic mobility and social activity among individuals with a lower extremity amputation. Arch Phys Med Rehabil 2001; 82: 1238–1244. 11552197 10.1053/apmr.2001.25079

[35] Mandel A , Paul K , Paner R *et al*. Balance confidence and activity of community‐dwelling patients with transtibial amputation. J Rehabil Res Dev 2016; 53: 551–560. 27898155 10.1682/JRRD.2015.03.0044

[36] Molsted S , Kusk L , Esbensen SM *et al*. Motives and barriers to exercise training during hospitalization in patients with type 2 diabetes: a cross‐sectional study. Int J Environ Res Public Health 2022; 19: 1035. 10.3390/ijerph19031035 PMC8834091 35162066

[37] Tang JH , Wang B , Chow JLJ *et al*. Improving postoperative mobilisation rates in patients undergoing elective major hepatopancreatobiliary surgery. Postgrad Med J 2021; 97: 239–247. 33184138 10.1136/postgradmedj-2020-138650

[38] Gama Lordello GG , Gonçalves Gama GG , Lago Rosier G , Viana PADC , Correia LC , Fonteles Ritt LE . Effects of cycle ergometer use in early mobilization following cardiac surgery: a randomized controlled trial. Clin Rehabil 2020; 34: 450–459. 31994405 10.1177/0269215520901763

[39] Stover G , Prahlow N . Residual limb pain: an evidence‐based review. NeuroRehabilitation 2020; 47: 315–325. 32986622 10.3233/NRE-208005

[40] Evans AG , Chaker SC , Curran GE *et al*. Postamputation residual limb pain severity and prevalence: a systematic review and meta‐analysis. Plast Surg (Oakv) 2022; 30: 254–268. 35990396 10.1177/22925503211019646 PMC9389065

[41] Bautmans I , Njemini R , De Backer J *et al*. Surgery‐induced inflammation in relation to age, muscle endurance, and self‐perceived fatigue. J Gerontol: Series A 2009; 65A: 266–273. 10.1093/gerona/glp145 19808837

[42] Xu XY , Lu JL , Xu Q , Hua HX , Xu L , Chen L . Risk factors and the utility of three different kinds of prediction models for postoperative fatigue after gastrointestinal tumor surgery. Support Care Cancer 2021; 29: 203–211. 32337625 10.1007/s00520-020-05483-0

[43] Avlund K . Fatigue in older adults: an early indicator of the aging process? Aging Clin Exp Res 2010; 22: 100–115. 20440097 10.1007/BF03324782

[44] Scheffer AC , Schuurmans MJ , van Dijk N , van der Hooft T , de Rooij SE . Fear of falling: measurement strategy, prevalence, risk factors and consequences among older persons. Age Ageing 2008; 37: 19–24. 18194967 10.1093/ageing/afm169

[45] Birhanie G , Melese H , Solomon G , Fissha B , Teferi M . Fear of falling and associated factors among older people living in Bahir Dar City, Amhara, Ethiopia a cross‐sectional study. BMC Geriatr 2021; 21: 586. 34674654 10.1186/s12877-021-02534-x PMC8532299

[46] Schoppen T , Boonstra A , Groothoff JW , de Vries J , Göeken LN , Eisma WH . Physical, mental, and social predictors of functional outcome in unilateral lower‐limb amputees. Arch Phys Med Rehabil 2003; 84: 803–811. 12808530 10.1016/s0003-9993(02)04952-3

[47] Essop‐Adam A , Daynes E , Houghton JSM *et al*. Clinimetrics of performance‐based functional outcome measures for vascular amputees: a systematic review. Ann Phys Rehabil Med 2023; 66: 101756. 37276748 10.1016/j.rehab.2023.101756

[48] Madsen UR , Biesbjerg CB , Mikkelsen TB , Marsaa K , Olsen Zwisler AD , Vedste Aagaard T . Considerable gaps and differences in rehabilitation after major lower extremity amputations across regions and municipalities in Denmark—a national survey. Scand J Caring Sci 2023; 37: 595–607. 36727432 10.1111/scs.13144

