# Supplementary material for: Fatigue, fear of being mobilized and residual limb pain limit independent basic mobility and physiotherapy for patients early after major dysvascular lower extremity amputation: A prospective cohort study
Source: Geriatr Gerontol Int. 2024 Apr 10;24(5):470–6. doi: 10.1111/ggi.14874 (PMC11503576; doi:10.1111/ggi.14874)
Supplement: Supplementary file 1 — Table S1. Basic Amputee Mobility Score and planned Physiotherapy scoring sheet. [file GGI-24-470-s001.docx]

| **Supplementary table 1**. Basic Amputee Mobility Score and planned Physiotherapy scoring sheet | | | | | |
| --- | --- | --- | --- | --- | --- |
| **For a Basic Amputee Mobility Score (BAMS) of 0 or 1 point in activity 1-4 or if planned physiotherapy cannot be completed, 1 to 2 limiting factors are reported** | | | | | **Patient ID** |
| Postoperative day no. |  |  |  |  | Limiting Factors **First, ask the patient what they experience as a limiting factor.**  **Note no. in the box:**   1. Residual limb pain 2. Pain elsewhere 3. Fear of being mobilized 4. Fatigue 5. Nausea/vomiting 6. Acute cognitive dysfunction (delirium) 7. Other (note at the bottom of this page) 8. ***** If the patient is not offered physiotherapy   **Note the 0–2 point BAMS score followed by / 1 to 2 limiting factors** in prioritized order, if relevant.  For patients not able to define a limiting factor, the physiotherapist in charge chooses the one considered the most obvious.  **Verbal Rating Scala (VRS)**  0 = No pain  1 = Slight pain  2 = Moderate pain  3 = Severe pain  4 = Unbearable pain  X = Patient not able to participate  **Planned physiotherapy completed**  If Yes (Y) if not able to fully complete enter Partially (P) or No (N) followed by / 1 to 2 limiting factors |
| Date |  |  |  |  |  |
| Time of assessment | * | * | * | * |  |
| Assessment done by (physio initials) |  |  |  |  |  |
| **Residual limb pain (at rest), VRS 0-4 point** |  |  |  |  |  |
| **1. From supine lying in bed to sitting on the edge of the bed and back,**  **BAMS 0-2 point** | **/** | **/** | **/** | **/** |  |
| **2. From sitting on the edge of the bed to wheelchair and back, BAMS 0-2 point** | **/** | **/** | **/** | **/** |  |
| **3. Wheelchair mobility, BAMS 0-2 point** | **/** | **/** | **/** | **/** |  |
| **4. From a chair/wheelchair to standing and back, BAMS 0-2 point** | **/** | **/** | **/** | **/** |  |
| **Residual limb pain during mobilization from bed to chair, VRS 0-4 point** |  |  |  |  |  |
| **No. of persons assisting in mobilization** |  |  |  |  |  |
| **Planned physiotherapy completed, Y-P-N** | **/** | **/** | **/** | **/** |  |
| **Pain assessment:** The patient is first asked if they experienced pain in the amputated leg/stump. If yes, ask further about the VRS categories, i.e., is it mild, moderate, severe, or worst pain imaginable that is experienced. Points (0-4) are only used to indicate the level of pain. | | | | |  |

**Other factors:** _______________________________________________________________________________________________________________________________________________________________________________________________________________________________________________________________________________________
