# Supplementary material for: Fatigue, fear of being mobilized and residual limb pain limit independent basic mobility and physiotherapy for patients early after major dysvascular lower extremity amputation: A prospective cohort study
Source: Geriatr Gerontol Int. 2024 Apr 10;24(5):470–6. doi: 10.1111/ggi.14874 (PMC11503576; doi:10.1111/ggi.14874)
Supplement: Supplementary file 2 — Table S2. Secondary limitations for not achieving an independent Basic Amputee Mobility Score (BAMS) of 2 points for each of the 4 BAMS activities and for partially or not completed planned physiotherapy on three consecutive days after major lower extremity amputation, N = 60. [file GGI-24-470-s002.docx]

| **Supplementary table 2.** Secondary limitations for not achieving an independent Basic Amputee Mobility Score (BAMS) of 2 points for each of the 4 BAMS activities and for partially or not completed planned physiotherapy on 3 consecutive days after major lower extremity amputation, N=60 | | | | | | | | | | | | |
| --- | --- | --- | --- | --- | --- | --- | --- | --- | --- | --- | --- | --- |
| **Secondary limitations for not achieving a Basic Amputee Mobility Score of 2 points per activity** | | | | | | | | | | | | |
|  | **PTDay1** | | | | **Day2** | | | | **Day3** | | | |
| **Limiting factor** | **A1** | **A2** | **A3** | **A4** | **A1** | **A2** | **A3** | **A4** | **A1** | **A2** | **A3** | **A4** |
|  | **(n=8)** | **(n=10)** | **(n=2)** | **(n=9)** | **(n=7)** | **(n=9)** | **(n=5)** | **(n=6)** | **(n=5)** | **(n=6)** | **(n=4)** | **(n=6)** |
| **Residual limb pain** | 3 (37.5) | 3 (30) | 1 (50) | 1 (11.1) | 3 (42.9) | 3 (33.3) | 1 (20) | 1 (16.7) | 1 (20) | 1 (16.7) | 1 (25) | 1 (16.7) |
| **Pain elsewhere** | - | - | - | - | - | - | - | - | - | - | - | - |
| **Fear of being mobilized** | 1 (12.5) | 2 (20) | - | 1 (11.1) | 1 (14.3) | 2 (22.2) | - | 1 (16.7) | - | - | - | - |
| **Fatigue** | 4 (50) | 5 (50) | 1 (50) | 6 (66.7) | 2 (28.6) | 2 (22.2) | 2 (40) | 3 (50) | 2 (40) | 3 (50) | 1 (25) | 2 (33.3) |
| **Nausea/vomiting** | - | - | - | - | - | - | - | - | 1 (20) | 1 (16.7) | 1 (25) | 1 (16.7) |
| **Acute cognitive dysfunction** | - | - | - | - | - | 1 (11.1) | 1 (20) | - | - | - | - | - |
| **Other*** | - | - | - | 1 (11.1) | 1 (14.3) | 1 (11.1) | 1 (20) | 1 (16.7) | 1 (20) | 1 (16.7) | 1 (25) | 2 (33.3) |
| **Secondary limitations for partially or not completed planned physiotherapy** | | | | | | | | | | | | |
| **Limiting factor** | **PTDay1 (n=4)** | | | | **Day2 (n=3)** | | | | **Day3 (n=2)** | | | |
| **Residual limb pain** | 1 (25) | | | | - | | | | - | | | |
| **Pain elsewhere** | 1 (25) | | | | - | | | | - | | | |
| **Fear of being mobilized** | 1 (25) | | | | 1 (33.3) | | | | 1 (50) | | | |
| **Fatigue** | 1 (25) | | | | - | | | | - | | | |
| **Nausea/vomiting** | - | | | | - | | | | - | | | |
| **Acute cognitive dysfunction** | - | | | | 1 (33.3) | | | | - | | | |
| **Other*** | - | | | | 1 (33.3) | | | | 1 (50) | | | |
| PTDay1 and Day2-3: the first day with physiotherapy and the following two days. Data are number N (percentages). A1-A4: Activity 1-4. A1. from supine lying in bed to sitting on the edge of the bed and back, A2. from sitting on the edge of the bed to a chair/wheelchair and back, A3. indoor wheelchair mobility and A4. from a chair/wheelchair to standing and back.  *Other: not motivated, fell out of bed/waiting for a doctor, and no comment specified. | | | | | | | | | | | | |
